# Measuring Creative Self-Efficacy: An Item Response Theory Analysis of the Creative Self-Efficacy Scale

**DOI:** 10.3389/fpsyg.2021.678033

**Published:** 2021-07-01

**Authors:** Amy Shaw, Melissa Kapnek, Neil A. Morelli

**Affiliations:** ^1^Department of Psychology, Faculty of Social Sciences, University of Macau, Avenida da Universidade, Macau, China; ^2^Berke Assessment, Atlanta, GA, United States; ^3^Codility, San Francisco, CA, United States

**Keywords:** creativity, creative self-efficacy, personality, creative self-efficacy scale, item response theory

## Abstract

Applying the graded response model within the item response theory framework, the present study analyzes the psychometric properties of Karwowski’s creative self-efficacy (CSE) scale. With an ethnically diverse sample of US college students, the results suggested that the six items of the CSE scale were well fitted to a latent unidimensional structure. The scale also had adequate measurement precision or reliability, high levels of item discrimination, and an appropriate range of item difficulty. Gender-based differential item functioning analyses confirmed that there were no differences in the measurement results of the scale concerning gender. Additionally, openness to experience was found to be positively related to the CSE scale scores, providing some support for the scale’s convergent validity. Collectively, these results confirmed the psychometric soundness of the CSE scale for measuring CSE and also identified avenues for future research.

## Introduction

Defined as “the belief one has the ability to produce creative outcomes” (Tierney and Farmer, 2002, p. 1138), creative self-efficacy (CSE; Tierney and Farmer, 2002, 2011; Beghetto, 2006; Karwowski and Barbot, 2016) has attracted increasing attention in the field of creativity research. The concept of CSE originates from and represents an elaboration of Bandura’s (1997) self-efficacy construct. According to Bandura (1997), self-efficacy influences what a person tries to accomplish and how much effort she/he may exert on the process. As such, CSE reflects a self-judgment of one’s own creative capabilities or potential which, in turn, affects the person’s activity choice and effort and, ultimately, the attainment of innovative outcomes. Lemons (2010) even claimed that it is not the competence itself but the mere belief about it that matters. Therefore, CSE appears to be an essential psychological attribute for researchers to understand the exhibition and improvement of creative performance. Indeed, there has been empirical evidence supporting the motivational importance of CSE and its capability of predicting crucial performance outcomes in both educational and workplace contexts (e.g., Schack, 1989; Tierney and Farmer, 2002, 2011; Choi, 2004; Beghetto, 2006; Gong et al., 2009; Karwowski, 2012, 2014; Karwowski et al., 2013; Puente-Díaz and Cavazos-Arroyo, 2017).

Given the important role of CSE, having psychometrically sound assessments of this construct is critical. Responding to the call for more elaborate CSE measures (Beghetto, 2006; Karwowski, 2011), the Short Scale of Creative Self (SSCS; Karwowski, 2012, 2014; Karwowski et al., 2018) was designed to measure trait-like CSE and creative personal identity (CPI; the belief that creativity is an important element of self-description; Farmer et al., 2003) by asking respondents to indicate the degree to which they include the construct as part of who they are on a 5-point Likert scale. The SSCS is composed of 11 items with six items measuring CSE and five items measuring CPI; CSE is often studied together with CPI, but both of the CSE and CPI subscales can be used as stand-alone scales (Karwowski, 2012, 2014; Karwowski et al., 2018). Specifically, CSE is described by the following six statements on the SSCS: Item (3) “I know I can efficiently solve even complicated problems,” Item (4) “I trust my creative abilities,” Item (5) “Compared with my friends, I am distinguished by my imagination and ingenuity,” Item (6) “I have proved many times that I can cope with difficult situations,” Item (8) “I am sure I can deal with problems requiring creative thinking,” and Item (9) “I am good at proposing original solutions to problems” (Karwowski et al., 2018, p. 48).

Since its introduction, the CSE scale has attracted research attention and there is some validity evidence supporting its use. In the formal scale development and validation study based on a sample of *n* = 622 participants, Karwowski et al. (2018) found that the 6-item CSE scale had a very good internal consistency reliability estimate, consisted of one predominant factor (i.e., CSE) and showed good convergent validity with moderate to large correlations with other CSE measures, such as the brief measures proposed by Beghetto (2006) and Tierney and Farmer (2002). Other empirical studies that adopted this instrument also suggested that the scale possessed fairly good estimates of reliability and validity (e.g., Karwowski, 2012, 2014, 2016; Karwowski et al., 2013; Liu et al., 2017; Puente-Díaz and Cavazos-Arroyo, 2017; Royston and Reiter-Palmon, 2019; Qiang et al., 2020).

Despite its promise, few studies beyond those by the scale developers have been conducted to investigate psychometric soundness of the 6-item CSE scale in terms of reliability and validity. Moreover, although the CSE scale has been thoroughly examined in samples from Poland (e.g., Karwowski et al., 2018), it has not yet been investigated for its psychometric properties in the US sample. Finally, all psychometric studies of the CSE scale so far have relied on the classical test theory (CTT) approaches in lieu of more appropriate modern test theory or item response theory (IRT; Steinberg and Thissen, 1995; Embretson and Reise, 2000) approaches (see also Shaw, Elizondo, and Wadlington 2020; for a recent discussion of applying advanced IRT models).

This study thus attempts to remedy these issues. Within the validity framework established by the *Standards for Educational and Psychological Testing* (American Educational Research Association, American Psychological Association, and National Council on Measurement in Education, 2014), here we report on a psychometric evaluation of the CSE scale in a sample of the US college students using IRT analyses. Specifically, item quality, measurement precision or reliability, dimensionality, and relations to external variables are evaluated. To our knowledge, it is also the first study that applies IRT to investigate the latent trait and item-level characteristics, such as item difficulty and discrimination of the CSE scale. Differential item functioning (DIF) analysis was conducted to examine the equivalence of individual item functioning across two gender subgroups, given some empirical findings on gender differences (albeit weak and inconsistent) in CSE (higher self-rated CSE by males; Beghetto, 2006; Furnham and Bachtiar, 2008; Karwowski, 2009). Additionally, concurrent validity was examined *via* evaluating the relationships among the CSE scale scores and the Big Five personality traits that have been found to be linked to CSE positively or negatively (e.g., Karwowski et al., 2013).

## Materials and Methods

### Participants

A total of *n* = 173 undergraduates at a large public university in the southern United States participated in this study for research credits. Participants’ ages ranged from 18 to 24 years with an average age of 20.60 (*SD* = 0.80). Among these subjects, *n* = 101 (58.4%) were female and *n* = 72 (41.6%) were male. The most commonly represented majors in the sample were psychology (32.8%), other social sciences (28.1%), and engineering (23.2%). Based on self-declared demographic information, 38.5% were Hispanic/Latino, 26.3% were Caucasian, 20.7% were Asian, 11.2% were African-American, and 3.4% selected other for ethnicity; the sample was thus ethnically diverse.

### Study Procedure and Materials

After providing their written informed consent, participants completed a standard demographic survey in addition to the 6-item CSE scale (Karwowski, 2012, 2014; Karwowski et al., 2018) and the Ten-Item Personality Inventory (TIPI; Gosling et al., 2003) for Big Five personality. The TIPI is comprised of 10 items with each containing a pair of trait descriptors; each trait is represented by two items: one stated in a way that characterizes the positive end of the trait and the other stated in a way that characterizes the negative end. For the TIPI, participants were asked to rate the extent to which the pair of traits applies to him/her on a 7-point Likert scale (1 = strongly disagree; 7 = strongly agree). All the TIPI trait scales showed similar internal consistency estimates to those reported in other studies (Gosling et al., 2003; Muck et al., 2007; Romero et al., 2012; Łaguna et al., 2014; Azkhosh et al., 2019): Extraversion (*α* = 0.68, *ω* = 0.69), Agreeableness (*α* = 0.45, *ω* = 0.47), Conscientiousness (*α* = 0.51, *ω* = 0.52), Emotional Stability (*α* = 0.71, *ω* = 0.71), and Openness to Experience (*α* = 0.46, *ω* = 0.47). These values are relatively low according to the rule of thumb of *α* = 0.70 (Nunnally, 1978, p. 245) but considered reasonably acceptable for a scale of such brevity (Gosling et al., 2003; Romero et al., 2012). For the CSE scale, participants were asked to indicate the extent to which each of the statements describes him/her on a 5-point Likert scale (1 = definitely not; 5 = definitely yes). Therefore, possible total scores on the CSE scale could range from 6 to 30, with higher scores indicating greater CSE.

## Analyses and Results

All cases were included in the final analyses (*n* = 173). The unidimensional IRT analysis of the CSE scale was conducted in IRTPRO (Cai et al., 2011) using Samejima’s (1969, 1997) graded response model (GRM), a suitable IRT model for data with ordered polytomous response categories, such as Likert-scale survey data (Steinberg and Thissen, 1995; Gray-Little et al., 1997). In GRM, each item has a slope parameter and between-category threshold parameters (one less than the number of response categories). In the current analysis, each item had five ordered response categories and thus four threshold parameters. Typically, in IRT models, the latent trait scale (theta-axis) is set with the assumption that the sample group is from a normally distributed population (mean value = 0; standard deviation = 1). This also applies to the GRM in the current study, and therefore, a theta value of 0 represents average CSE and a theta value of −1.00, for example, suggests being one standard deviation below the average.

Table 1 lists the item parameter estimates for all six items together with their standard errors, which can be used to evaluate how each item performs. The slope or item discrimination parameter *a*(s) reflects the strength of the relationship between the item response and the underlying construct, which indicates how fast the probabilities of responses change across the trait level (i.e., CSE). Generally, items with higher slope parameters provide more item information. The slopes for the six items were all higher than 1.00, and the associated standard errors ranged from 0.21 to 0.31, indicating a satisfactory degree of discriminating power for the six items (Steinberg and Thissen, 1995; Cai et al., 2011). The category threshold or item boundary location parameters *b*(s) reflect the points on the latent trait scale (theta-axis) at which a respondent has a 50% probability of endorsing above the threshold; higher threshold parameters suggest the items are more difficult (i.e., requiring higher trait level to endorse). For example, looking at the first row of Table 1, one can see that for Item 1 (or Item 3 on the original SSCS scale: “I know I can efficiently solve even complicated problems”), a respondent with a trait level (theta value) of −1.15 (*b*_1_) has a 50% probability of endorsing “2 = *probably not*” or higher, with a trait level of −0.09 (*b*_2_) has a 50% probability of endorsing “3 = *possibly*” or higher, with a trait level of 0.71 (*b*_3_) has a 50% probability of endorsing “4 = *probably yes*” or higher, and with a trait level of 1.71 (*b*_4_) has a 50% probability of endorsing “5 = *definitely yes*.” As displayed in Table 1, all threshold parameter estimates ranged from −3.42 to 2.33, indicating that the items provided good measurement in terms of item difficulty across an adequate range of the underlying trait (i.e., CSE).

**Table 1:** Slope and category threshold parameter estimates for all six Items.

Item	Label	Slope (*a*)	*s.e.*	Threshold 1 (*b*_1_)	*s.e.*	Threshold 2 (*b*_2_)	*s.e.*	Threshold 3 (*b*_3_)	*s.e.*	Threshold 4 (*b*_4_)	*s.e.*
1	SSCS 3	1.84	0.31	−1.15	0.18	−0.09	0.11	0.71	0.14	1.71	0.24
2	SSCS 4	1.18	0.21	−2.41	0.41	−0.87	0.20	0.30	0.15	2.23	0.38
3	SSCS 5	1.40	0.24	−2.45	0.37	−1.39	0.23	−0.22	0.13	1.55	0.25
4	SSCS 6	1.15	0.22	−3.42	0.62	−1.34	0.27	−0.16	0.16	1.72	0.31
5	SSCS 8	1.28	0.24	−1.41	0.26	−0.30	0.15	1.07	0.21	2.33	0.39
6	SSCS 9	1.65	0.28	−1.81	0.27	−0.59	0.15	0.40	0.13	1.48	0.22

*Note. SSCS = Short Scale of Creative Self*.

One assumption underlying the application of unidimensional IRT models is that a single psychological continuum (i.e., the latent trait) accounts for the covariation among the responses. The assumption of unidimensionality and the model fit could be evaluated simultaneously by examining the presence of local dependence (LD) among pairs of the scale items. Referring to excess covariation between item pairs that could not be accounted for by the single latent trait in the unidimensional IRT model, LD implies that the model is not adequately capturing all item covariances. The standardized chi-square statistic (standardized LD *χ*^2^; Chen and Thissen, 1997) was used for the evaluation of LD; standardized LD *χ*^2^ values of 10 or greater are generally considered noteworthy. Goodness of fit of the GRM was evaluated using the *M*_2_ statistic and the associated RMSEA value (Cai et al., 2006; Maydeu-Olivares and Joe, 2006). As presented in Table 2, the largest standardized LD *χ*^2^ value was 2.9 (less than 10) so there was no indication of LD among the six items and thus no violation of unidimensionality for pairs of items. Goodness of fit indices also demonstrated that the unidimensional GRM had satisfactory fit [*M*_2_ (305) = 438.22, *p* < 0.001; *RMSEA* = 0.04]. Besides, we looked at the summed-score-based item fit statistics [*S-χ^2^* item-level diagnostic statistics; Orlando and Thissen, 2000, 2003; also see Roberts (2008) for a discussion of extensions] for further evaluation (significant values of *p* indicate lack of item fit). As presented in Table 3, all probabilities were above 0.05 so that there was no item flagged as potentially problematic or misfitting.

**Table 2:** Marginal fit (*χ*^2^) and standardized LD *χ*^2^ statistics.

Item	Label	Marginal *χ*^2^	Standardized LD *χ*^2^ of item pairs				
			Item 1	Item 2	Item 3	Item 4	Item 5
1	SSCS 3	0.2	–				
2	SSCS 4	0.2	1.3	–			
3	SSCS 5	0.2	2.3	2.5	–		
4	SSCS 6	0.2	0.2	2.7	−0.4	–	
5	SSCS 8	0.2	0.1	2.9	1.0	1.8	–
6	SSCS 9	0.1	1.6	1.0	0.0	2.1	−0.2

*Note. SSCS = Short Scale of Creative Self*.

**Table 3:** S-*χ*^2^ item-level diagnostic statistics.

Item	Label	*χ*^2^	*d.f.*	Probability
1	SSCS 3	48.13	41	0.21
2	SSCS 4	50.17	42	0.18
3	SSCS 5	50.90	40	0.12
4	SSCS 6	43.59	39	0.28
5	SSCS 8	46.37	43	0.34
6	SSCS 9	31.37	40	0.83

*Note. SSCS, Short Scale of Creative Self*.

**Figure 1:**
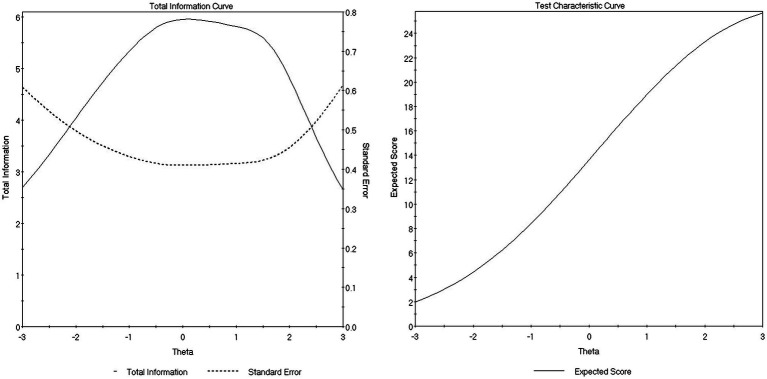
Test information curve **(left panel)** and test characteristic curve **(right panel)**.

In Figure 1, the left and right panels present the test information curve (together with its corresponding standard errors line) and test characteristic curve, respectively. The test information curve was created by adding together all six-item information curves. The test information curve describes varying measurement precision provided at each trait level (IRT information is the expected value of the inverse of the error variances for each estimated value of the latent trait) and estimates how well the construct is measured at all levels of the underlying trait, thus showing how well the measure functions as a whole across the latent trait continuum for the model. Generally, more psychometric information equals greater measurement precision (with lower error). As graphically illustrated in the left panel of Figure 1, the test information curve peaks in the middle (total information for the entire scale is approximately 6.00 in that range), indicating that the test provided the most information (or smallest standard errors of measurement) in the middle (and slightly-to-the-right) range of trait level estimates (where most of the respondents are located) but little information for those at extremely low or high ends of CSE (i.e., theta values outside the range of −3.00 to 3.00 along the construct continuum). The calculated *Expected A Posteriori*-based marginal reliability value was 0.82. Thus, the CSE scale in the current study appeared to work well (and was the most informative/sensitive) for differentiating individuals in the middle and middle-to-high of the trait range (where most people reside). The test characteristic curve, as displayed in the right panel, presents the expected values of the summed observed scores of the entire scale as a function of theta values (i.e., the CSE trait levels). For instance, the zero-theta value corresponds to the expected summed score of 13.63. The close-to-linear curve for values of CSE on the continuum between −2.00 and 2.00 suggests that the summed observed scores were a good approximation of the latent trait scores estimated in GRM.

Differential item functioning detection was performed using the Mantel test (Mantel, 1963). No evidence of DIF was found between male and female respondents [DIF contrasts were below 0.50, Mantel-Haenszel probabilities for all items were above 0.05, and thus, there was no indication of a statistically significant difference of item functioning across gender subgroups; the effect sizes of all items were also classified as small/negligible according to the ETS delta scale (Zieky, 1993; Sireci and Rios, 2013; Shaw et al., 2020)], suggesting item fairness of the CSE scale regarding gender.

In addition, concurrent validity was examined by evaluating the Big Five personality correlates of the CSE total scores using Pearson’s correlation. Replicating part of the findings in past work (e.g., Jaussi et al., 2007; Silvia et al., 2009; Karwowski et al., 2013), openness to experience was found to be positively related to CSE (*r* = 0.23, *p* < 0.01). Other traits, however, were not found to be related to CSE in the current sample: Extraversion (*r* = 0.13, *p* = 0.09), Agreeableness (*r* = 0.08, *p* = 0.30), Conscientiousness (*r* = 0.10, *p* = 0.19), and Emotional Stability (*r* = 0.06, *p* = 0.43).

## Discussion

In the present study, we aimed to better understand the psychometric properties of the 6-item CSE scale (Karwowski, 2012, 2014; Karwowski et al., 2018). Applying GRM in IRT, we found that the items were well fitted to a single latent construct model, providing support for the scale as a unidimensional measure of CSE. This finding is in line with previous studies using more traditional but less sophisticated approaches for categorical response data in CTT (e.g., Karwowski et al., 2018). The IRT analyses also suggested high levels of item discrimination, an appropriate range of item difficulty, as well as satisfactory measurement precision primarily suitable for respondents near average CSE. Furthermore, the gender-based DIF detection confirmed that there was no gender DIF for the CSE items, so that any score difference between the two gender subgroups on the CSE scale could be attributable to meaningful differences in the underlying construct (i.e., CSE), making the CSE scale, a useful instrument for studying gender and CSE. Regarding correlations with relevant criteria, CSE positively related to openness to experience, exhibiting some convergence validity. Collectively, these results provided initial evidence supporting the psychometric soundness of the CSE scale for measuring CSE among the US college students.

Notably, the CSE scale is a domain-general self-rating scale. Despite the ongoing debate on whether creativity and creative self-concept shall be better measured as domain-general or domain-specific constructs, Pretz and McCollum (2014) suggested that there is an association between CSE and the belief to be creative on both domain-general and more domain-specific self-ratings, albeit the varying effect sizes that might be dependent on domain specificity. Moreover, in spite of some doubts concerning self-rated creativity as a valid and useful measure of actual creativity (Reiter-Palmon et al., 2012), there is research evidence suggesting that subjective and objective ratings of creativity tended to be positively correlated (Furnham et al., 2005); a growing body of empirical work in the CSE literature has also elucidated that self-judgments about one’s creative potential could serve as a crucial motivational/volitional factor driving actions that may lead to creative performance (Tierney and Farmer, 2002, 2011; Farmer et al., 2003; Beghetto, 2006; Karwowski and Barbot, 2016). At the very least, self-assessments of creativity could be a nice complement to other types of creativity assessments in cases where objective performance metrics are unavailable for research.

Several limitations of the current study are also worth noting. First, the relatively small sample size makes all interpretations of the results subject to suspicion, given the fact that a GRM was applied and each item had five response categories—the large amount of possible response patterns definitely benefits from having a larger sample size which would allow for a more convincible conclusion. Second, although an ethnically diverse sample was used, it was a convenience college student sample, and thus, the results should be considered within the context, and any generalization of the findings to other populations shall be done with caution. That said, further research with larger and more representative college student sample or samples from other populations (e.g., working adults, graduate students, and high school students) is warranted. Third, even though no gender difference in the CSE scale scores was observed in the current study, this finding should be interpreted with caution given the fact that the sample was slightly predominated by females. Also with the sample consisting of a majority of students from psychology or other social sciences majors, the results regarding the absence of gender differences in the current sample require further examination. Studies have not converged on the relationship between CSE and gender, but in a study by Kaufman (2006), males self-reported greater creativity than females in areas of science and sports, whereas females self-reported greater creativity than males in domains of social communication and visual artistic factors. Therefore, it is likely that the characteristics of our current sample (predominated by females and mostly in social sciences) limited the capacity of the study to detect potential gender differences in CSE. Future research using more gender-balanced samples with diverse academic majors is recommended. Last, the inherent limitation of the personality scale used in the current study may have contributed to the smaller size of the CSE-openness correlation compared to findings in other studies that used more comprehensive personality measures (e.g., Furnham et al., 2005; Karwowski et al., 2013; Pretz and McCollum, 2014). Although the TIPI has been widely used and is characterized by satisfactory correlations with other personality measures, this brief personality scale only consists of 10 items (two for each trait) which often inevitably results in lower internal consistencies and somewhat diminished validities (Gosling et al., 2003; Romero et al., 2012).

In sum, by demonstrating satisfactory item-level discriminating power, an appropriate range of item difficulty, good item fit and functioning, adequate measurement precision or reliability, and unidimensionality for the CSE scale, this study provided support for its internal construct validity. The positive CSE-openness relationship finding also provided some evidence for the scale’s convergent validity. Future research may further assess the predictive validity of the CSE scale on outcome measures, preferably in comparison with other less elaborate measures of CSE in the literature. Based on the results of the current work, the 6-item CSE scale could be a useful and appropriate CSE measurement tool for researchers and practitioners to conveniently incorporate in studies. It is also our hope that this study together with past work will facilitate even more efforts to develop, validate, and refine instruments for CSE.

## Data Availability Statement

The data analyzed in this study are subject to the following licenses/restrictions: Restrictions apply to the availability of these data, which were used under license for this study. Data are available from the authors upon reasonable request. Requests to access these datasets should be directed to AS, amyshaw@um.edu.mo.

## Ethics Statement

Ethical review and approval was not required for the study on human participants in accordance with the local legislation and institutional requirements. The patients/participants provided their written informed consent to participate in this study.

## Author Contributions

All authors listed have made a substantial, direct and intellectual contribution to the work and approved it for publication.

### Conflict of Interest

NM was employed by company Codility Ltd.

The remaining authors declare that the research was conducted in the absence of any commercial or financial relationships that could be construed as a potential conflict of interest.
